# Identification of a novel P2X7 antagonist using structure-based virtual screening

**DOI:** 10.3389/fphar.2022.1094607

**Published:** 2023-01-12

**Authors:** Gaia Pasqualetto, Marika Zuanon, Andrea Brancale, Mark T. Young

**Affiliations:** ^1^ School of Biosciences, Cardiff University, Cardiff, United Kingdom; ^2^ School of Pharmacy and Pharmaceutical Sciences, Cardiff University, Cardiff, United Kingdom; ^3^ Department of Organic Chemistry, University of Chemistry and Technology, Prague, Czechia

**Keywords:** P2X7, P2X4, virtual screen, structure-based drug design, antagonist

## Abstract

P2X4 and P2X7 receptors are ATP-gated ion channels, which play important roles in neuropathic and inflammatory pain, and as such they are important drug targets in diseases of inflammatory origin. While several compounds targeting P2X4 and P2X7 receptors have been developed using traditional high-throughput screening approaches, relatively few compounds have been developed using structure-based design. We initially set out to develop compounds targeting human P2X4, by performing virtual screening on the orthosteric (ATP-binding) pocket of a molecular model of human P2X4 based on the crystal structure of the *Danio rerio* receptor. The screening of a library of approximately 300,000 commercially available drug-like compounds led to the initial selection of 17 compounds; however, none of these compounds displayed a significant antagonist effect at P2X4 in a Fluo-4 ATP-induced calcium influx assay. When the same set of compounds was tested against human P2X7 in an ATP-stimulated Yo-Pro1 dye uptake assay, one compound (an indeno(1,2-b)pyridine derivative; GP-25) reduced the response by greater than 50% when applied at a concentration of 30 µM. GP-25 displayed an IC_50_ value of 8.7 μM at human P2X7 and 24.4 μM at rat P2X7, and was confirmed to be active using whole-cell patch clamp electrophysiology and not cytotoxic. Schild analysis suggested that mode of action of GP-25 was orthosteric. Screening of a further 16 commercially available analogues of GP-25 led to the discovery of five additional compounds with antagonist activity at human P2X7, enabling us to investigate the structure-activity relationship. Finally, docking of the R- and S-enantiomers of GP-25 into the orthosteric pocket of molecular models of human P2X4 and human P2X7 revealed that, while both enantiomers were able to make multiple interactions between their carboxyl moieties and conserved positively charged amino-acids in human P2X7, only the S-enantiomer of GP-25 was able to do this in human P2X4, potentially explaining the lack of activity of GP-25 at this receptor.

## 1 Introduction

P2X receptors are a family of seven ATP-gated ion channels, which have important functions in neurotransmission, pain and inflammation (Sheng and Hattori, 2022). The P2X4 and P2X7 subtypes in particular are involved in modulation of inflammation and chronic inflammatory pain. P2X4 receptors are involved in tactile allodynia following nerve damage (Tsuda et al., 2003), and P2X7 receptors are thought to modulate inflammatory responses in conditions including arthritis, Crohn’s disease and age-related macular degeneration, as well as being involved in cancer progression and mood disorders (Ren and Illes, 2022). Due to their involvement in inflammatory disease, P2X receptors are important drug targets (Dane et al., 2022) and many P2X4 and P2X7 antagonists have been discovered and developed, largely using high throughput screening approaches (Hernandez-Olmos et al., 2012; Ase et al., 2015; Müller and Namasivayam, 2022), but also by structure-based screening (Caseley et al., 2016; Zhao et al., 2021). The discovery of novel P2X4 receptor antagonists has been more of a challenge than for P2X7, and this may be because many antagonists (including BX430 and 5-BDBD for P2X4 (Fischer et al., 2004; Ase et al., 2015), and A740003, A804598, AZ10606120, GW791343, JNJ47965567, A438079 and AZ11645373 for P2X7 (Honore et al., 2006; Karasawa and Kawate, 2016; Allsopp et al., 2018; Bin Dayel et al., 2019)) bind to an allosteric pocket in the extracellular domain, which is smaller in P2X4 than P2X7 (Karasawa and Kawate, 2016).

Structure-based virtual screening is an efficient approach for the discovery of novel “hit” compounds with activity against a target protein. In this process, compounds are docked *in silico* into a crystal structure or molecular model. The quality of the docking is scored and then the top-scoring “hits” are selected for biological assay. If a library of commercially available drug-like molecules is used for the screen, the compounds can be purchased for assay without requiring any chemical synthesis. Crystal structures are now available for *Danio rerio* (zebrafish) P2X4 in complex with ATP (Hattori and Gouaux, 2012), human P2X3 in complex with ATP and the competitive antagonists A-317491 and TNP-ATP (Mansoor et al., 2016), and giant panda P2X7 in complex with allosteric antagonists (Karasawa and Kawate, 2016), as well as the cryoEM structure of full-length rat P2X7 in complex with ATP (McCarthy et al., 2019), giving a series of good starting points to develop molecular models of P2X receptors. Structure-based screening on the ATP-binding (orthosteric) pocket of P2X7 has also been used successfully, leading to the identification of hit compounds with selectivity for P2X7 over P2X4 (Caseley et al., 2016). A similar approach has also been used to discover P2X4 and P2X1 antagonists, although their potency was in the high micromolar range (Beswick et al., 2019).

In this work we used structure-based virtual screening on the orthosteric pocket of a molecular model of human P2X4 based upon the zebrafish P2X4 crystal structure (Hattori and Gouaux, 2012). We first validated our docking algorithms by docking ATP into the orthosteric pocket, then performed a virtual screen of the Specs library of commercially available drug-like compounds (Specs.net), selecting a total of 17 compounds for biological assay. None of the compound candidates displayed antagonist activity at human P2X4 in an ATP-induced calcium influx assay at 10 uM. However we identified one compound (GP-25; an indeno(1,2-b)pyridine derivative) with antagonist activity at.net both human and rat P2X7 in an ATP-induced dye uptake (YoPro-1) assay, with IC_50_ values of 8.7 μM at human P2X7 and 24.4 μM at rat P2X7. We confirmed the antagonist activity of GP-25 in whole-cell patch clamp experiments, and Schild analysis suggested orthosteric antagonism [although there was a reduction in peak response at the highest antagonist concentration tested (100 µM)]. Sixteen compound analogues to GP-25 were then purchased and tested on human and rat P2X7, leading to the identification of a further five molecules with antagonist activity and permitting the analysis of the structure-activity relationship. *In silico* docking simulations performed on the orthosteric pocket suggested that both the R- and S- enantiomers of GP-25 (as well as GP-47, one of the other analogues with antagonist activity) could bind to human P2X7, as opposed to only the S-enantiomer of GP-25 in human P2X4. Both the R- and S-enantiomers of GP-25 and GP-47 were able to occupy the P2X7 orthosteric pocket in a way that preserved key interactions between carboxyl groups and conserved positively charged amino-acids in the orthosteric pocket, but only the S-enantiomer of GP-25 was able to do this in human P2X4. This gave a potential molecular explanation for the lack of activity of GP-25 that we observed at human P2X4. In summary, our work describes both the structure-based discovery of a novel orthosteric human P2X7 antagonist, and an analysis of the molecular basis for the differential potencies observed at different receptor subtypes.

## 2 Materials and methods

### 2.1 Development of 1321N1 cells stably expressing human P2X4, human and rat P2X7 receptors

1321N1 astrocytoma cells and HEK-293 cells were cultured (at 37°C, 5% CO_2_) in Dulbecco’s modified Eagle’s medium (DMEM/F-12 with Glutamax) supplemented with 10% foetal bovine serum (FBS) and 200 unit/ml of penicillin and streptomycin antibiotics (Fisher Scientific). For stable cell line generation, cells were transfected using FuGene HD Transfection Reagent (Promega) according to the manufacturer’s protocol with plasmids encoding either human P2X4 with a C-terminal (His)_10_ tag (Young et al., 2008), human P2X7 with a C-terminal EYMPME tag (kindly provided by R.A. North) or rat P2X7 C-terminally tagged with a GFP-(His)_8_ tag, generated by PCR amplification of the coding sequence for rat P2X7 from a pcDNA-based expression vector (Young et al., 2007), adding XhoI and EcoRI sites at the 5′- and 3′- ends respectively, followed by restriction digestion and ligation into a zebrafish P2X4-GFP expression vector based on pEGFP-C1 (kindly provided by Eric Gouaux; Kawate and Gouaux, 2006) to replace the zebrafish P2X4 coding region with rP2X7. Transfected clones were grown in DMEM/F-12 with Glutamax medium supplemented with G-418 (Geneticin®, Fisher Scientific), 600 μg/ml and 150 μg/ml during selection and maintenance respectively. Each clone was tested for P2X4 or P2X7 receptor expression by Western blot and clones, which displayed receptor expression were assessed for functionality using the Ca^2+^ influx assay (P2X4) or the Yopro-1 uptake assay (P2X7). HEK-293 cells stably expressing human P2X7 (Adinolfi et al., 2010) were a kind gift from Elena Adinolfi.

### 2.2 Homology modelling and ligand docking

*In silico* simulations were performed on a MAC pro 2.80 GHz Quad-Core Intel Xeon running Ubuntu 12.04 LTS. Graphical representations were generated with MOE (Molecular Operating Environment, 2014.09; Chemical Computing Group ULC, Canada). Structure files retrieved from the RCSB Protein Data Bank (http://www.rcsb.org) as PDB file format were used as template to build P2X4 [PDB ID 4DW1 (Hattori and Gouaux, 2012)] and P2X7 [PDB ID 6U9W (McCarthy et al., 2019)] receptor homology models using MOE 2014.09, single template mode and default settings with AMBER12:EHT force field, including the crystallized ATP molecules for the induced-fit mode. Each model was checked visually and *via* the Rampage Server, Molprobity (Lovell et al., 2003) and PROCHECK (Laskowski et al., 1993) to exclude gross errors in protein geometry. For validation, ATP coordinates in the crystal structure were used to set the centroid coordinated for a box of 10 Å^3^ grid used in the docking. FlexX 2.1.3 with default parameters (Rarey et al., 1996), PLANTS (Korb et al., 2006) and Glide Standard Precision (https://www.schrodinger.com/products/glide) simulations were used for the docking. 20 aco ants (for the explorative docking algorithm) and standard parameters were set for simulations with PLANTS. 10 or more poses generated in each simulation were visually inspected.

### 2.3 Structure-based virtual screening and docking

Preparation of compound structure was performed with the LigPrep module (https://www.schrodinger.com/products/ligprep), using the Specs library of commercially available compounds (∼300,000 entries; Specs.net). Briefly, hydrogens were added and possible ionization states were generated for pH 7.0 ± 2.0 using the OPLS_2005 forcefield; possible tautomers were generated for each molecule as well as all possible enantiomers for structures with non-specified chiralities. The center of the binding region was defined by either manual selection of the pocket residues or by selecting the superposed ligand. Ligands were initially docked using the Glide High throughput Screening (HTVS) protocol, the top 15% best scored poses were re-docked with the Glide Standard Precision protocol, and then rescored (without performing ulterior docking) using the Glide Extra Precision (XP) protocol, FlexX and PLANTS (in parallel). A consensus score from all 3 re-scorings was calculated (Bassetto et al., 2013), and poses that ranked best according to Glide XP, FlexX and PLANTS—were selected for visual inspection. Docking of GP-25 and analogues were performed on the human P2X7 and human P2X4 models using Glide XP protocol using a grid appropriate for 14 Å^3^ ligand length, with crystallized ATP coordinates as grid centroid. Poses were visually inspected before final selection of compounds for assay. Criteria in the choice of ligands included i) ability to entertain multiple H-bond or ionic interactions with key residues in the ATP binding pocket and ii) at least a partial overlap between the docked ligand conformation and the superposed adenine of the ATP. Additional interactions were also a favorable criteria.

### 2.4 Fluo4 calcium influx assay

Cells were plated the day before assay into poly-lysine-coated 96-well plates at 40–65,000 cells/ml. To load cells with calcium-sensitive fluorescent dye, culture medium was replaced with modified Ringer’s buffer (140 mM NaCl, 10 mM HEPES, 10 mM Glucose, 1 mM MgCl_2_, 1 mM CaCl_2_, 2.5 mM KCl, .5% BSA, pH = 7.4) containing 2.6 μM FLUO4-AM (Fisher Scientific) and 250 μM Probenecid (Sigma), and cells were incubated for 20–30 min. Immediately prior to assay, buffer was replaced with fresh modified Ringer’s buffer containing 500 μM Probenecid. Compounds were dissolved in DMSO at 1,000× the required assay concentration prior to diluting in assay buffer and the final DMSO concentration did not exceed .1%. A Fluoroskan Ascent FL plate reader (Fisher Scientific) equipped with a solution dispenser and an appropriate filter pair (excitation: 485 nm, emission: 538 nm) was used to record 5 s baseline followed by P2X4-mediated calcium influx measurements for 20–25 s after ATP stimulation (20 μL injection of solutions dissolved in modified Ringer’s buffer). The amplitude of ATP responses was calculated as ΔF/F0, where ΔF = F1 − F0, subtracting the fluorescent background as suggested by Bootman et al. (2013).

### 2.5 Yopro-1 uptake assay

Cells were plated as for the calcium influx assay (Section 2.4). Culture medium was replaced with low divalent cation containing extracellular solution (ECS-LD; 147 mM NaCl, 10 mM HEPES, 13 mM Glucose, .2 mM CaCl_2_, 2 mM KCl. pH = 7.4 in MilliQ water) and 5 μM Yopro-1. Compounds were dissolved in DMSO at 1,000× the required assay concentration prior to diluting in assay buffer and the final DMSO concentration did not exceeded .1%. The baseline was measured with a BMG Clariostar instrument for 5–10 cycles, followed by addition of ATP (or BzATP) and recording for at least 30 further cycles (approx. 1 min per data point). The collected responses were normalized to the first data-point recorded after ATP addition. The initial gradient of the dye uptake curve was used to calculate the response to ATP.

### 2.6 Patch-clamp electrophysiology

Whole-cell patch-clamp traces were recorded at room temperature using patch pipettes pulled from borosilicate glass (World Precision Instruments, Sarasota, FL, United States). Pipettes were filled with internal solution (IS) containing (in mM): 145 NaCl, 10 EGTA and 10 HEPES, with the pH adjusted to 7.3 with NaOH and had resistances of 3–5 MΩ. Cells were constantly perfused in a bath with ECS-LD. Currents were recorded at a holding membrane potential of −60 mV using an Axon Instruments Axopatch Multiclamp 700A amplifier and Digidata 1322A A/D interface (Molecular Devices, Sunnyvale, CA, United States). ATP working solutions were prepared fresh and solutions with compounds had a final concentration of DMSO equal or lower than .1% (.1% DMSO was included as vehicle control). ATP applications were made at 90-s intervals. Solutions were applied to patch-clamped cells with the help of a rapid perfusion system (RSC-160, Biologic, Claix, France), allowing solution exchange times in the range 20–100 m.

### 2.7 Cell viability assay

To assess hit compound toxicity, CellTiter-Blue Cell Viability Assay (Promega, Southampton, United Kingdom) was used as recommended by the manufacturer on not transfected HEK-293. Cells were seeded in 96-well plates (100 μL at a concentration of 30–35,000 cells/ml) in culture media and a day later media was replaced with 100 µL fresh media containing 30 µM of tested compounds (10 µM of A740003). After 24 h incubation at 37 °C, 5% CO_2_, 20 µL of CellTiter Blue reagent was added to each well and incubated for 3 h at 37°C. Fluorescence was then measured with a BMG Clariostar using excitation/emission wavelengths of 560/590 nm. Data were normalized to vehicle control-treated cells (.1% DMSO).

### 2.8 Data analysis and statistics

Data processing and data analysis were carried out using Graphpad Prism version 6 for Mac. Figures are presented as mean ± SEM. Statistical significance of any difference observed between samples was calculated through one-way ANOVA followed by Dunnett’s multiple comparisons test with a single pooled variance (control, unless otherwise specified) or, when comparing only two sets of data, using an unpaired t-test with Welch’s correction. When comparing data obtained from multiple independent experiments (N), each dataset was normalized to the mean value obtained for the vehicle control before pooling data. A non-linear regression (curve fit) with four parameters was applied to determine any dose-response correlation and the presence of any inhibition trend during the compound testing (curve-fit constraints were applied to normalized data). Y = Bottom + (Top-Bottom)/(1 + 10^((LogEC_50_-X)*Hill Slope)) Where: X: agonist (or antagonist) concentrations expressed as logarithmic scale. Y: response measurements (raw or normalized value) This formula was also used for inhibition curve fitting by substituting the value of EC_50_ with IC_50_.

## 3 Results

### 3.1 Virtual screening and compound selection

We generated a molecular model of human P2X4 based on the crystal structure of zebrafish P2X4 bound to ATP (Hattori and Gouaux, 2012). Three docking algorithms were validated—later employed in the virtual screening (PLANTS, FlexX and Glide SP)—by docking ATP in the model. In each simulation the docked pose of ATP was similar to that observed for ATP in the crystal structure (superposed, shown in grey lines in Supplementary Figure S1); in the dock obtained using PLANTS (Supplementary Figure S1A) the adenine ring was translated (positioned less deep in the pocket), with FlexX (Supplementary Figure S1B) the phosphate chain conformation was different (the *ß* and gamma phosphates in the docking occupied the *a* and *ß* positions in the model derived from the crystal structure), and with Glide SP (Supplementary Figure S1C) there was the greatest similarity. A protein-ligand interaction fingerprint (PLIF) showed comparable interactions in the zebrafish P2X4 crystal structure (4DW1) and our three docking simulations (Supplementary Figure S1D). In our docking simulations we observed some additional interactions with Leu-188 (surface contact), Lys-190 (sidechain hydrogen bond acceptor and ionic attraction), Leu-214 (sidechain hydrogen bond donor and surface contact) and Ser-289 (sidechain and backbone hydrogen bond acceptor). Our results suggested that each algorithm was capable of replicating the binding pose of ATP in the orthosteric pocket, but as Glide performed the best, this was used as the main algorithm for subsequent virtual screening.

A library of commercially available drug-like compounds (Specs, 2022) with approximately 300,000 molecules was docked *in silico* into the orthosteric pocket of our human P2X4 model, selecting a total of 17 compounds for biological assay (Table 1).

**TABLE 1:** List of compounds identified by virtual screening in the P2X4 ATP-binding pocket (numbered 1–17) and GP-25 analogues (numbered 18–33).

ID	Structure	Specs ID	MW	% Inhibition at hP2X7	% Inhibition at rP2X7
1	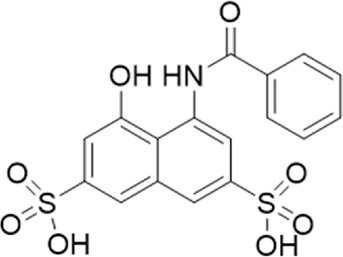	AE-641/00786016	423.42	41	n.d
2	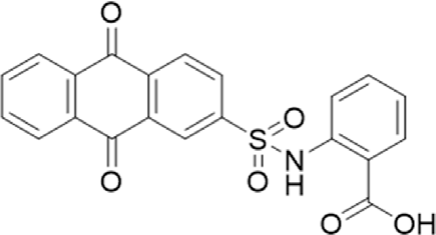	AE-641/42133418	407.40	38	n.d
3	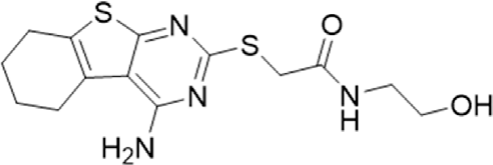	AF-399/40634562	338.45	—	n.d
4	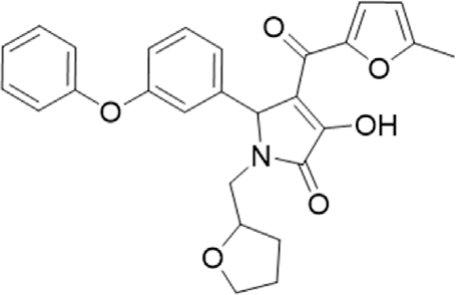	AF-399/41900709	459.50	<10	n.d
5	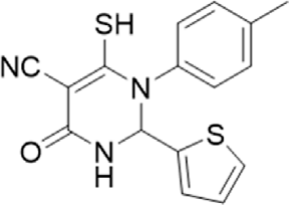	AF-399/42810490	327.43	—	n.d
6	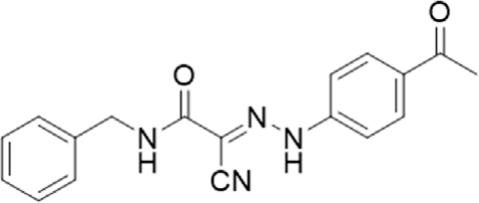	AF-407/33312043	320.35	<10	n.d
7	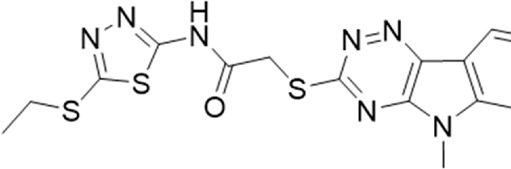	AG-205/36698032	417.54	n.d	n.d.
8	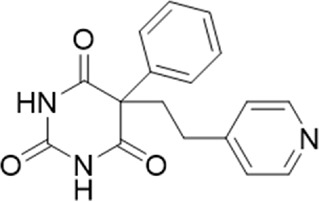	AG-670/36765017	309.32	—	n.d
9 (GP-25)	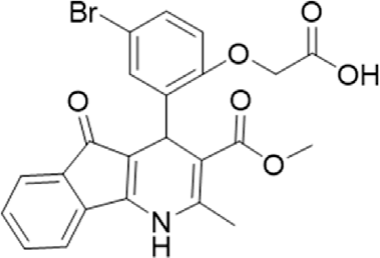	AH-487/14758206	484.30	∼75	47
10	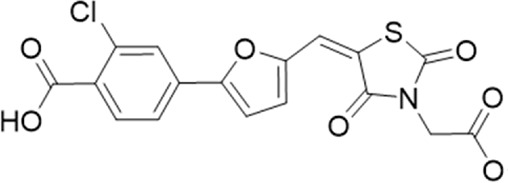	AH-487/40935627	435.84	—	n.d
11	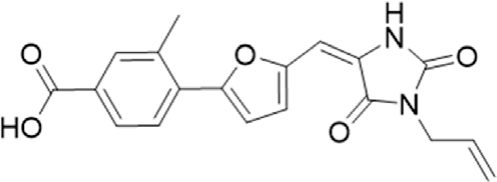	AH-487/41955121	352.34	25	n.d
12	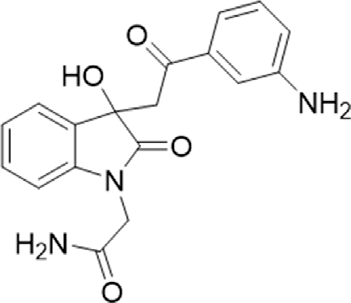	AK-778/41182449	339.35	12	n.d
13	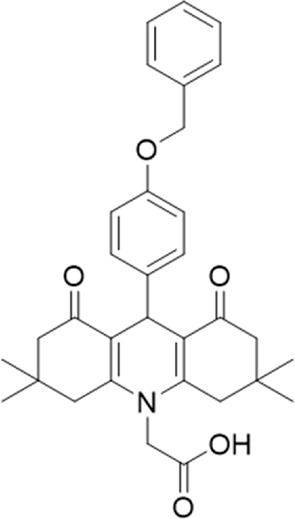	AN-988/40787450	513.63	29	n.d
14	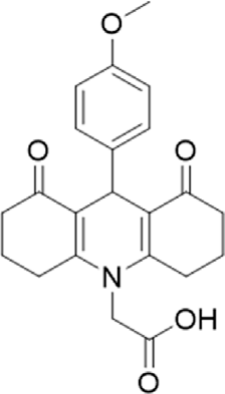	AN-988/40787687	381.43	—	n.d
15	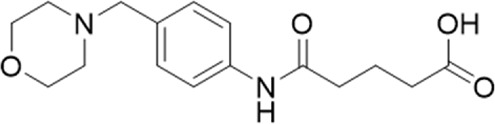	AO-080/43441580	306.36	11	n.d
16	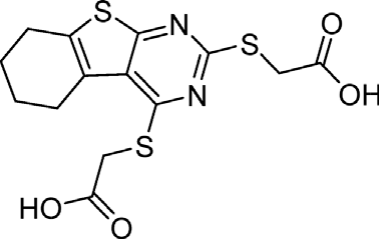	AO-476/41610193	370.47	<10	n.d
17	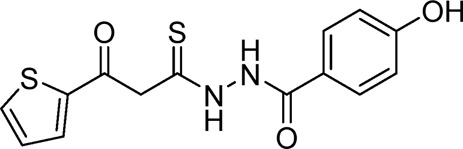	AO-990/15068055	321.38	<10	n.d.
18	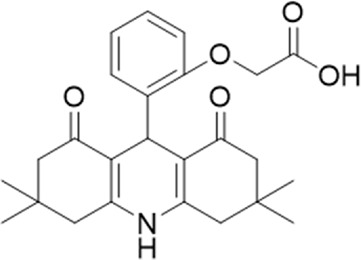	AK-968/15604330	423.51	—	—
19	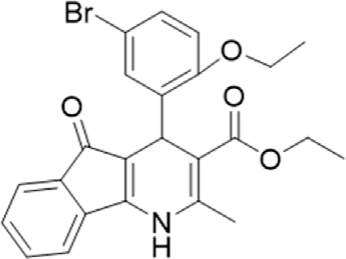	AH-487/15150556	468.35	—	13
20 (GP-47)	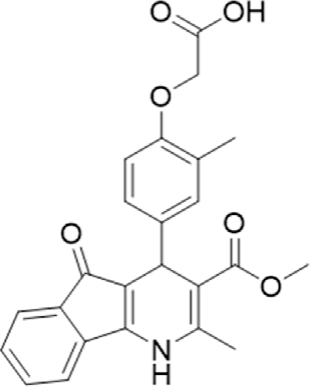	AH-487/14757970	439.85	77	—
21	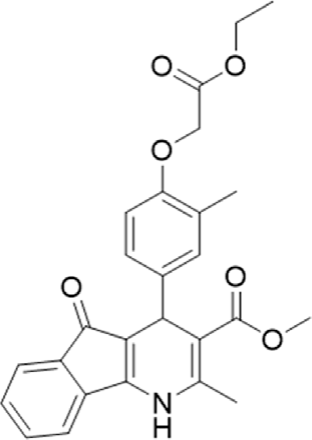	AH-487/14758229	433.46	<10	—
22 (GP-50)	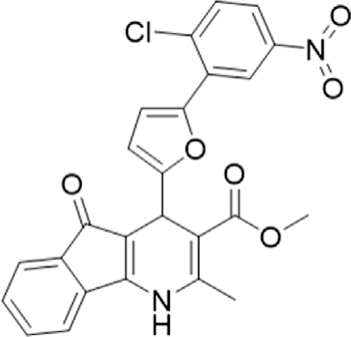	AG-690/40638872	476.87	69	—
23	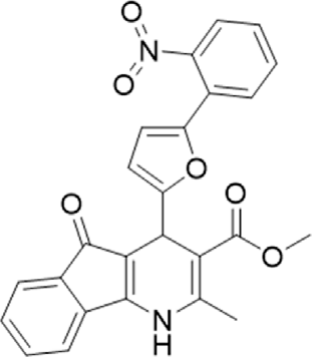	AG-690/40638873	442.43	30	—
24	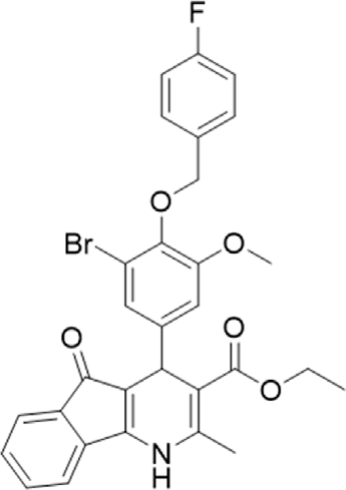	AN-988/40667255	578.43	12	16
25	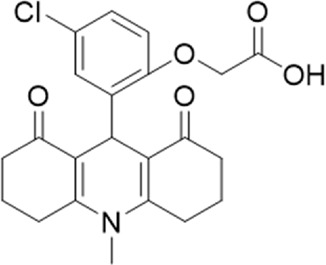	AM-879/42012121	415.87	20	—
26	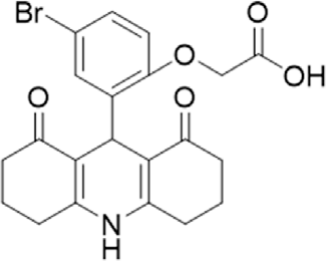	AM-879/42012369	446.3	22	—
27	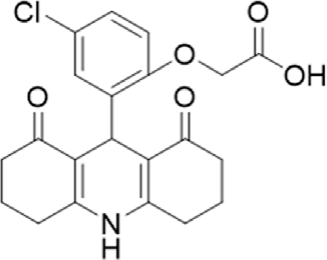	AM-879/42012458	401.84	16	—
28	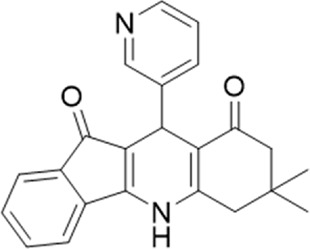	AJ-292/42032071	356.42	32	—
29	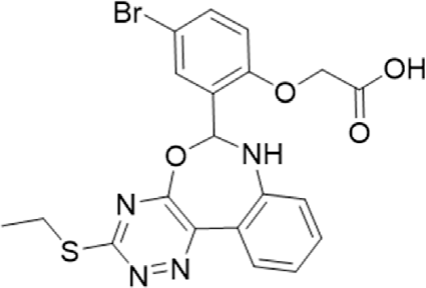	AK-968/11109126	489.35	20	—
30	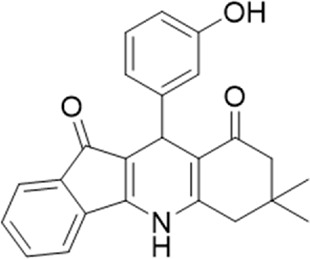	AJ-292/14827035	371.43	27	—
31	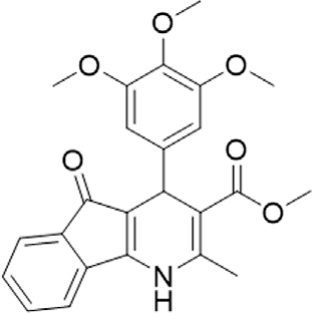	AH-487/14758041	421.45	29	<10
32 (GP-66)	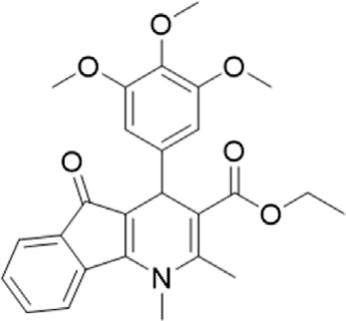	AK-454/40961396	449.5	55	19
33	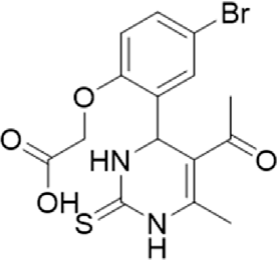	AN-655/41064474	399.27	18	16

Specs IDs, identification number according to the supplier (specs.net). MW, molecular weight (g/mol). Approx. % inhibition at 30 μM at human P2X7 and rat P2X7. —for compound displaying increased responses vs. control. n.d, not determined.

### 3.2 Identification of a P2X7 antagonist among the screened compounds

**FIGURE 1:**
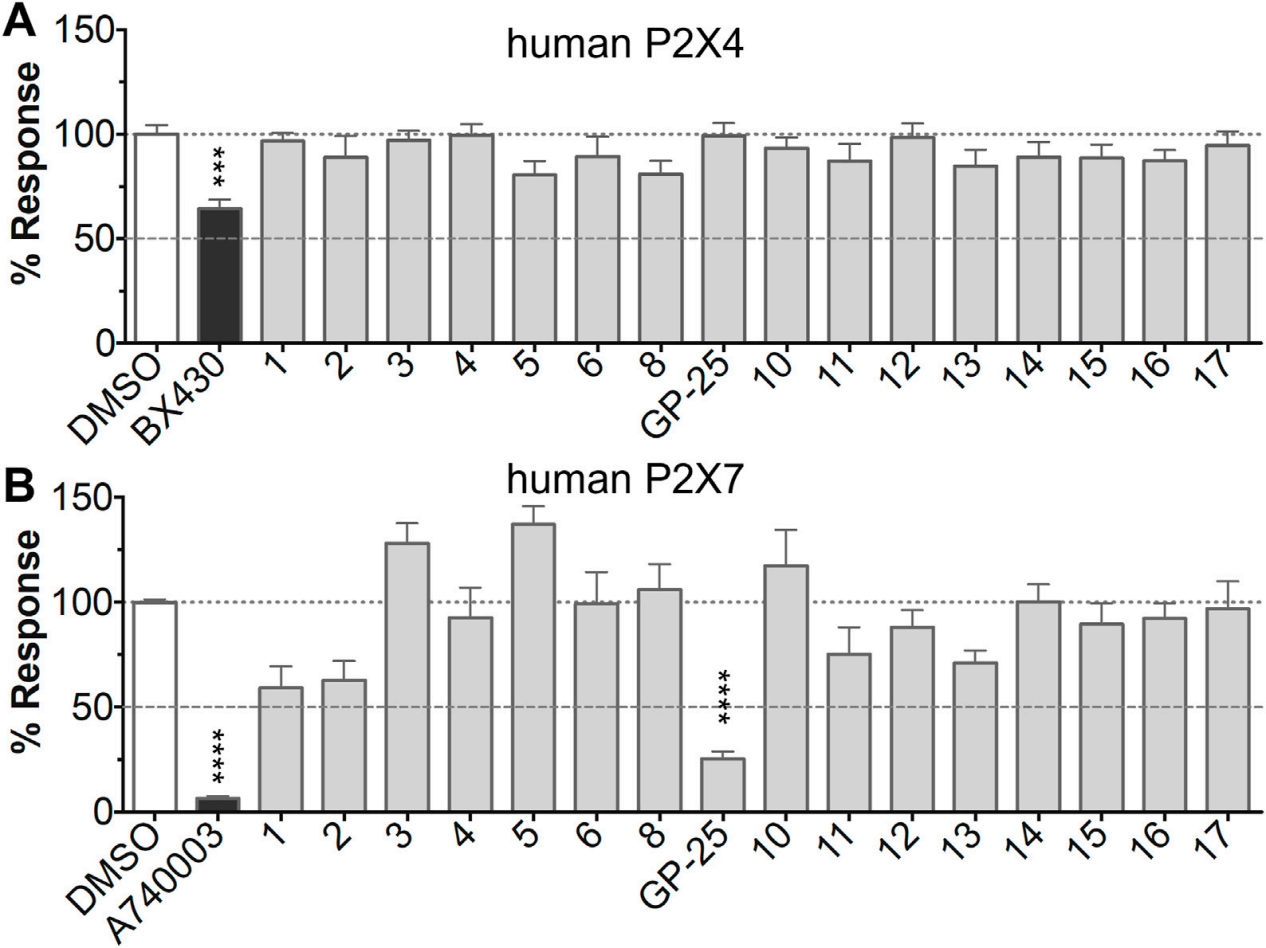
Initial screening of compounds at human P2X4 and human P2X7. **(A)** Normalized % response of 1321N1 astrocytoma cells stably transfected with human P2X4 to 1.2 µM ATP (Fluo-4 calcium uptake assay) incubated in the absence (DMSO) or presence of either 2 µM BX-430 or 10 µM Specs compound (1–16; GP-25 is compound 9). **(B)** Normalized % response of 1321N1 astrocytoma cells stably transfected with human P2X7 to 300 µM ATP (YoPro-1 dye uptake assay) incubated in the absence (DMSO) or presence of either .1 µM A740003 or 30 µM Specs compound. The final concentration of DMSO did not exceed .1%. Asterisks represent significant differences from control (DMSO) as measured by one-way ANOVA and Dunnet’s test (***, *p* ≤ .001; ****, *p* ≤ .0001). Data represents two or more independent experiments (*n* = 3–4 each).

The antagonist activity of the 17 compounds (applied at 10 µM) was assayed initially on 1321N1 astrocytoma cells stably expressing human P2X4 receptors by measuring the ATP-induced (applied at 1.2 µM) increase in Fluo-4 fluorescence due to calcium influx *via* P2X4, using 2 µM BX430 as a positive control (Figure 1A). Compound 7 was excluded from biological assay due to very low solubility. None of the compounds showed a statistically significant reduction in response, suggesting no antagonist activity at human P2X4. The same compounds were then assayed at a single concentration of 30 µM (we initially expected that, having designed the compounds to bind to P2X4, they would likely be less potent at P2X7, and need to be applied at a higher concentration to observe an effect) on 1321N1 astrocytoma cells stably expressing human P2X7. The ATP-induced rate of uptake of YoPro-1 dye was measured upon application of at 300 µM ATP, using .1 µM A740003 as a positive control (Figure 1B). Several compounds showed at least 25% reduction in response (Table 1) and, strikingly, one compound (GP-25; (4-bromo-2-(3-(methoxycarbonyl)-2-methyl-5-oxo-4,5-dihydro-1H-indeno(1,2-b)pyridine-4-yl)phenoxy)acetic acid) inhibited the response by approximately 74.6%, and was taken forward for further analysis.

### 3.3 Characterization of GP-25 antagonism at P2X7

**FIGURE 2:**
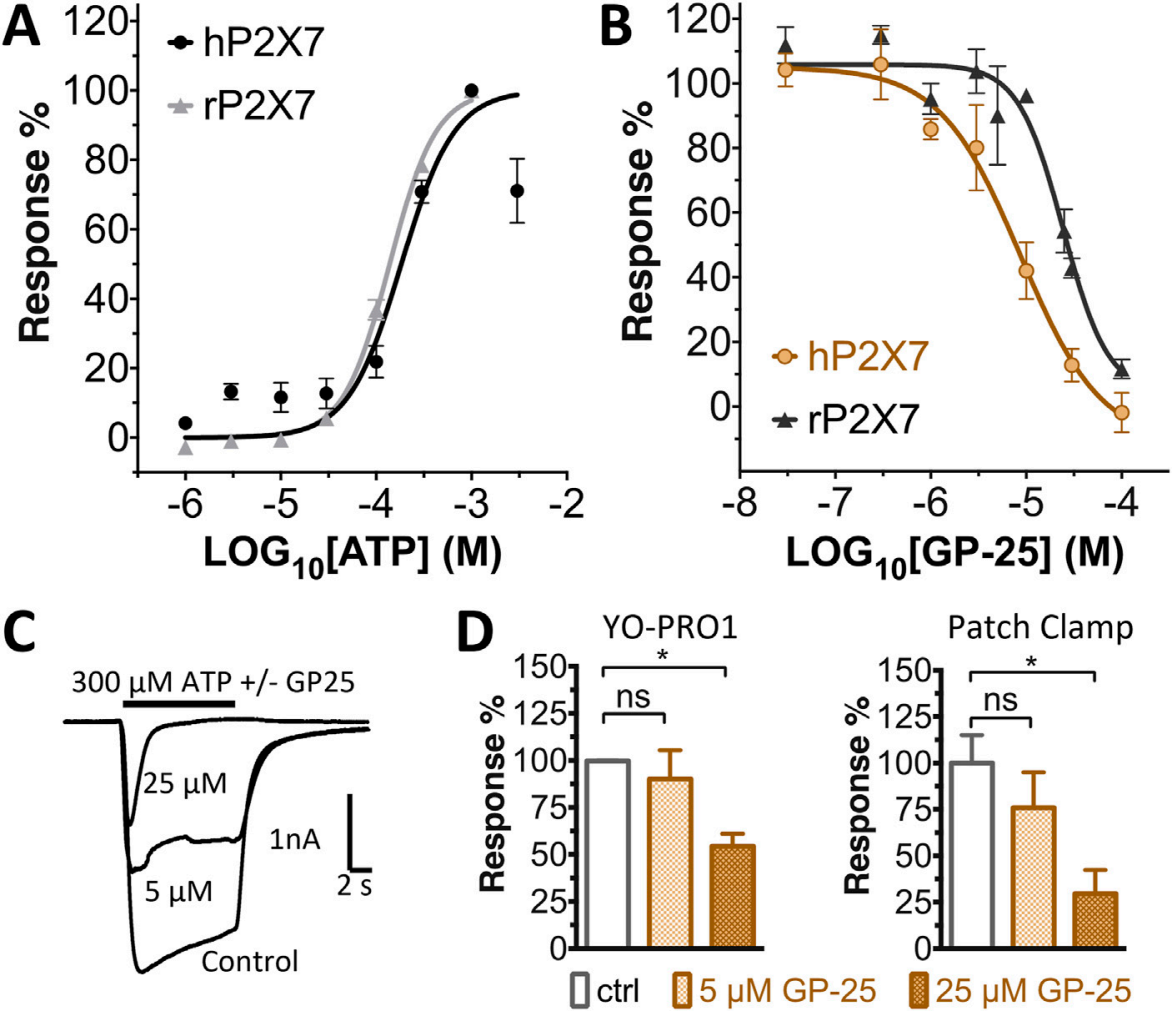
Functional characterization of GP-25. **(A)** ATP concentration-response curves (YoPro-1 assay) for human P2X7 (black line, circles) and rat P2X7 (grey line, triangles). Estimated EC_50_ values were 183.5 and 138.3 µM for human and rat P2X7, respectively. **(B)** GP-25 concentration-inhibition curves (YoPro-1 assay) for human P2X7 (brown line, circles) and rat P2X7 (black line, triangles) (300 µM ATP). Estimated IC_50_ values were 8.7 μM at human P2X7 and 24.4 μMat rat P2X7. **(C)** Representative traces for human P2X7 showing inhibition of ATP-evoked currents by GP-25 (concentrations indicated) in whole-cell patch clamp. **(D)** Summary of GP-25 inhibition data from YoPro-1 (left) and patch clamp (right) experiments. *; *p* ≤ .05 (One-way ANOVA). ns; non-significant. YoPro-1 assay data represents two independent experiments (*n* = 3–5 each); electrophysiology *n* = 5–7 for each GP-25 concentration.

**FIGURE 3:**
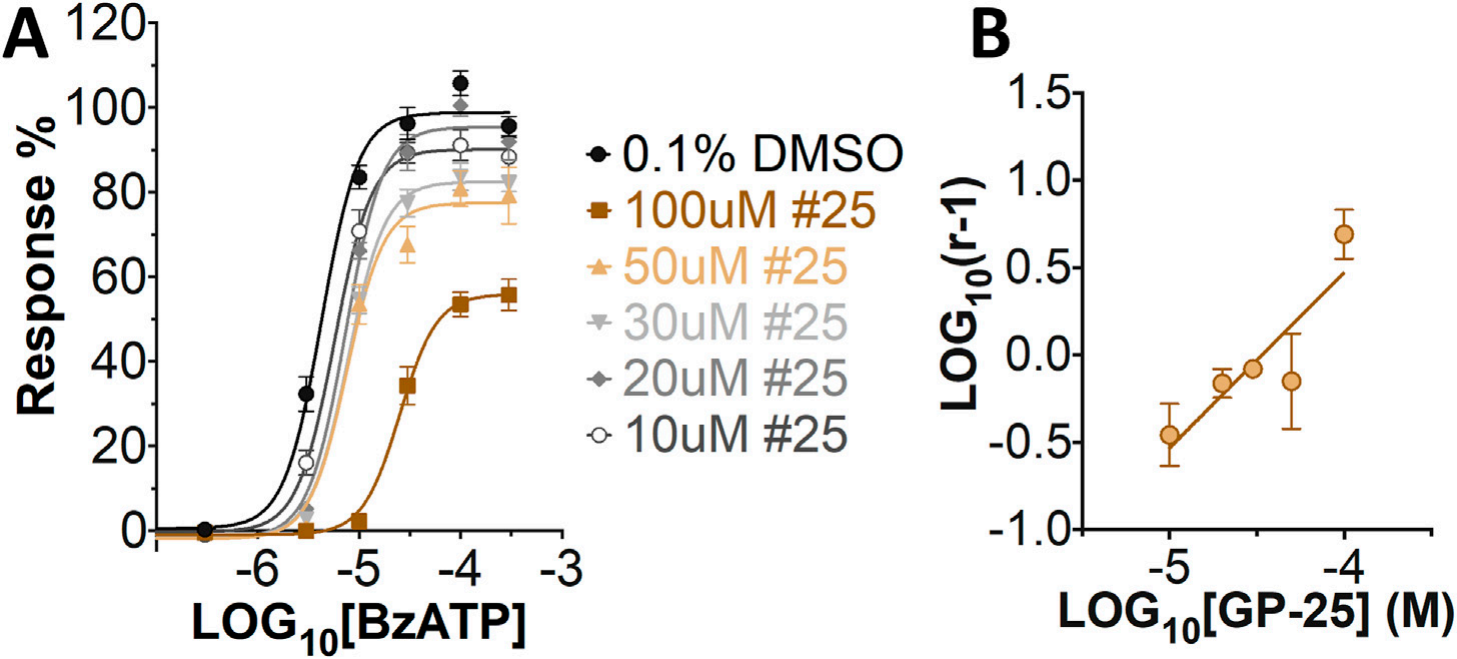
GP-25 is an orthosteric P2X7 antagonist. **(A)** BzATP concentration-response curves in increasing concentrations of GP-25 (compared to control; .1% DMSO). Curves are fitted without constraints (simple 4-parameter fitting). **(B)** Schild plot of the relationship between GP-25 concentration and the dose ratio. The equation for the line of best fit was Y = 1.004*X + 4.489; R2= .5785. Slope was 1.004 ± .2584; not significantly different from the theoretical value of 1 at *p* = .05. Data represents three independent experiments (*n* = 3 each).

Concentration-response curves for both human and rat P2X7 receptors were constructed using the ATP-induced YoPro-1 uptake assay, and EC_50_ values of 183.5 and 138.3 µM were determined for human and rat P2X7, respectively (Figure 2A). Concentration-inhibition curves were constructed for GP-25 at both human and rat P2X7 using the YoPro-1 uptake assay (Figure 2B; 300 µM ATP), and IC_50_ values of 8.7 μM at human P2X7 and 24.4 μM at rat P2X7 were determined, demonstrating moderate selectivity of GP-25 for human P2X7 over the rat orthologue. To confirm antagonist action in an independent assay, whole-cell patch clamp experiments were performed on human embryonic kidney (HEK-293) cells stably expressing rat P2X7-GFP (Figure 2C), and a concentration-dependent inhibition was observed when GP-25 was pre-applied for 2 min (300 µM ATP application). A statistically significant reduction in P2X7 activity was observed upon application of 25 µM GP-25 in both the YoPro-1 and patch clamp assays confirming GP-25 activity in the low micromolar range (Figure 2D). In order to confirm GP-25 as a competitive (orthosteric) antagonist, BzATP concentration-response curves (ranging between 300 μM and .03 µM) were constructed for rat P2X7-GFP in the YoPro-1 uptake assay in the presence of increasing concentrations of GP-25 (Figure 3A). Maximal responses were observed in all except the highest GP-25 concentration (100 µM). Schild analysis, plotting the logarithm of the ratio of apparent EC_50_ values ((antagonist/control) − 1) against the logarithm of GP25 concentration (Figure 3B) gave a straight line with a slope not significantly different than 1 (at *p* = .05 as determined by one-sample t-test against the theorical value 1; 3 biological replicates), indicative of competitive (orthosteric) antagonism.

### 3.4 Activities of GP-25 analogues at human and rat P2X7

**FIGURE 4:**
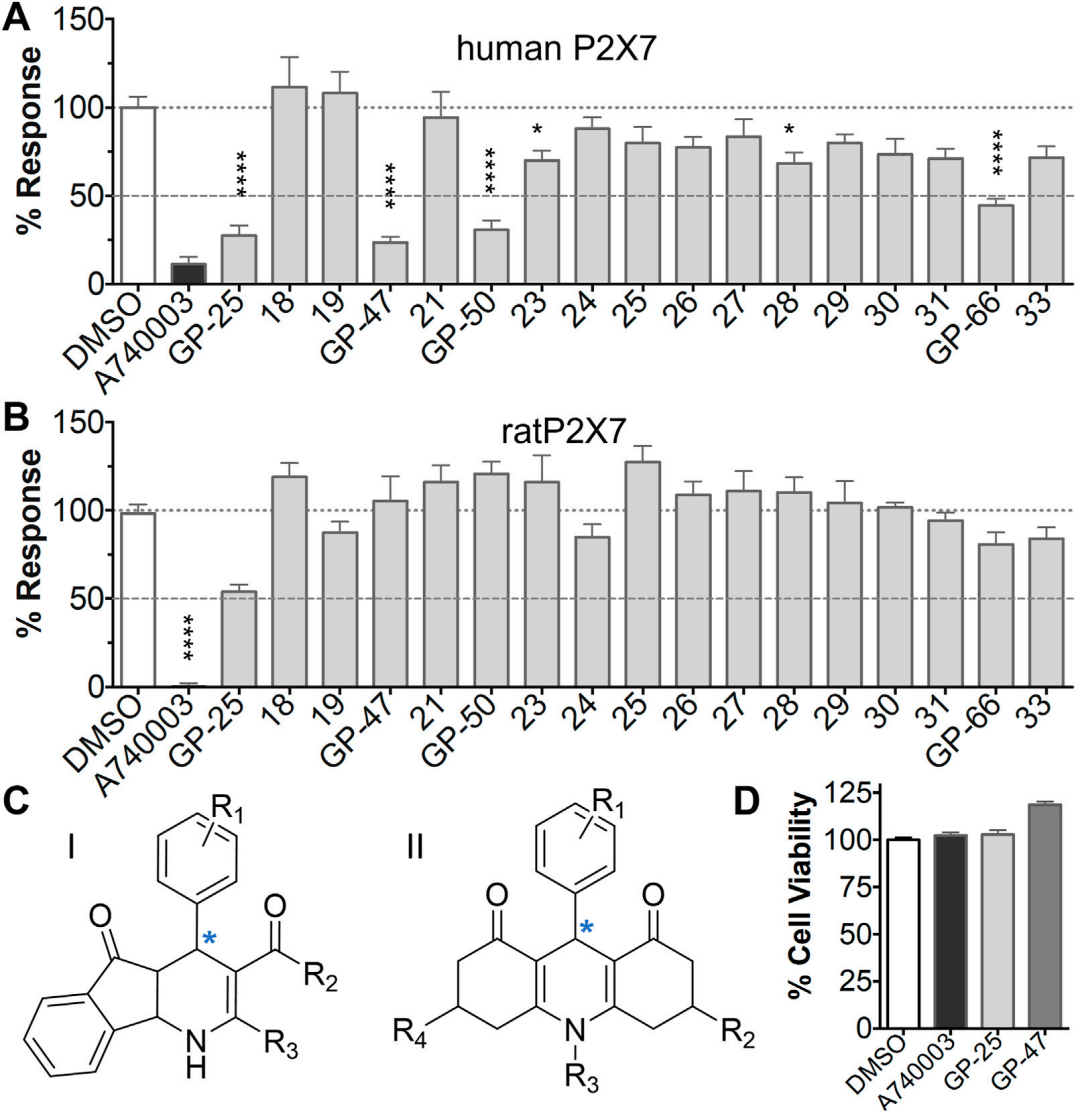
Activity of GP-25 analogues at human and rat P2X7. **(A,B)** Normalized % response of HEK cells transfected with either human P2X7 **(A)** or rat P2X7-GFP **(B)** to 300 µM ATP (YoPro-1 dye uptake assay) incubated in the absence (DMSO) or presence of either .1 µM A740003 or 30 µM Specs compound (18–33; GP-25 is compound 9, GP-47 is compound 20, GP-50 is compound 22 and GP-66 is compound 32). (*, *p* ≤ .05; ****, *p* ≤ .0001). Data represents two or more independent experiments (*n* = 3–4 each). **(C)** GP-25 analogue common chemical scaffolds. Scaffold I: R1, halogen, methoxyl-, methoxycarbonyl- and/or hydroxyl-. Compound 28 presents a pyridinyl- instead of the phenyl-. R2, methoxyl-, ethoxyl-. or cyclized ketone. R3, methyl-. Compound 28 presents a pyridinyl-. instead of the phenyl-. GP-66 has an N-methyl substitution on the indeno(1,2-b)pyridine moiety. Scaffold II: R1, halogen, methoxyl-, methoxycarbonyl- and/or hydroxyl-. R2 and R4, -H, dimethyl-. R3, -H, methyl-. Chiral center marked with a blue star. **(D)**. HEK cell viability (CellTitre Blue assay) following a 24 h incubation with either .1% DMSO (vehicle control) or 10 μM A740003, or 30 µM GP-25 or GP-47.

A small library of 16 commercially available compounds was selected according to the presence of the same scaffold as GP-25, (70% similarity, Tanimoto index) and/or presence of at least one functional group we hypothesized could contribute to GP-25 activity (e.g. carboxylic acid, halogen substituent to the aromatic ring, ester, etc.) (Compounds 18–33; Table 1). When human P2X7-expressing cells were incubated with 30 µM of compounds (Figure 4A), several GP-25 analogues caused a statistically significant reduction in ATP-induced dye uptake (300 μM ATP, approx. EC_70_ measured in our experiments), including GP-47, GP-50, Compound 23, Compound 28 and GP-66. None of these compounds displayed antagonist activity at rat P2X7 (Figure 4B and summarized in Table 1). Although the number of compounds tested was limited by their availability *via* a commercial source, the numerous ‘hits’ allowed for a basic structure-relationship (SAR) analysis. 13 out of 16 compounds presented either scaffold I or scaffold II—Scaffold I being the one shared among most of the compounds that showed moderate or good activity (Figure 4C). A methoxyl acetate as substituent (R1) on the phenyl ring (Scaffold I) appears to be important for compound activity, as substitution with a methoxyl group completely abolished the activity (as for Compound 19; Figure 4A and Table 1). Methoxyl acetate as either an ortho- or para-substituent (R1) was tolerated, as were halogens in the meta-position in the phenyl ring (R1), both moieties improving activity in Scaffold I. The introduction of a furan linker between the dihydro-1H-indeno(1,2-b)pyridine-3-carboxylate and the phenyl moiety (scaffold very similar to that of Scaffold I; Figure 4C) was permitted without loss of activity provided that there was of a halogen in the ortho- and a nitro-group in the para-position of the phenyl moiety (GP-50). However, we observed that GP-50 was less soluble than GP-47 or GP-25. Compounds bearing Scaffold II (Compound 13, 14, 18, 25, 26, 27) did not show activity over 25% at 30 μM, indicating that the substitution of the carboxylate to a cyclic ketone is not favored.

Finally, GP-25 and GP-47 were tested in a cytotoxicity assay showing that a 24-h incubation did not significantly reduce cell viability in HEK-293 wild-type (Figure 4D). As GP-50 showed low solubility, it was excluded from the cell viability assay.

### 3.5 Docking of GP-25 and GP-47 in the orthosteric pocket

**FIGURE 5:**
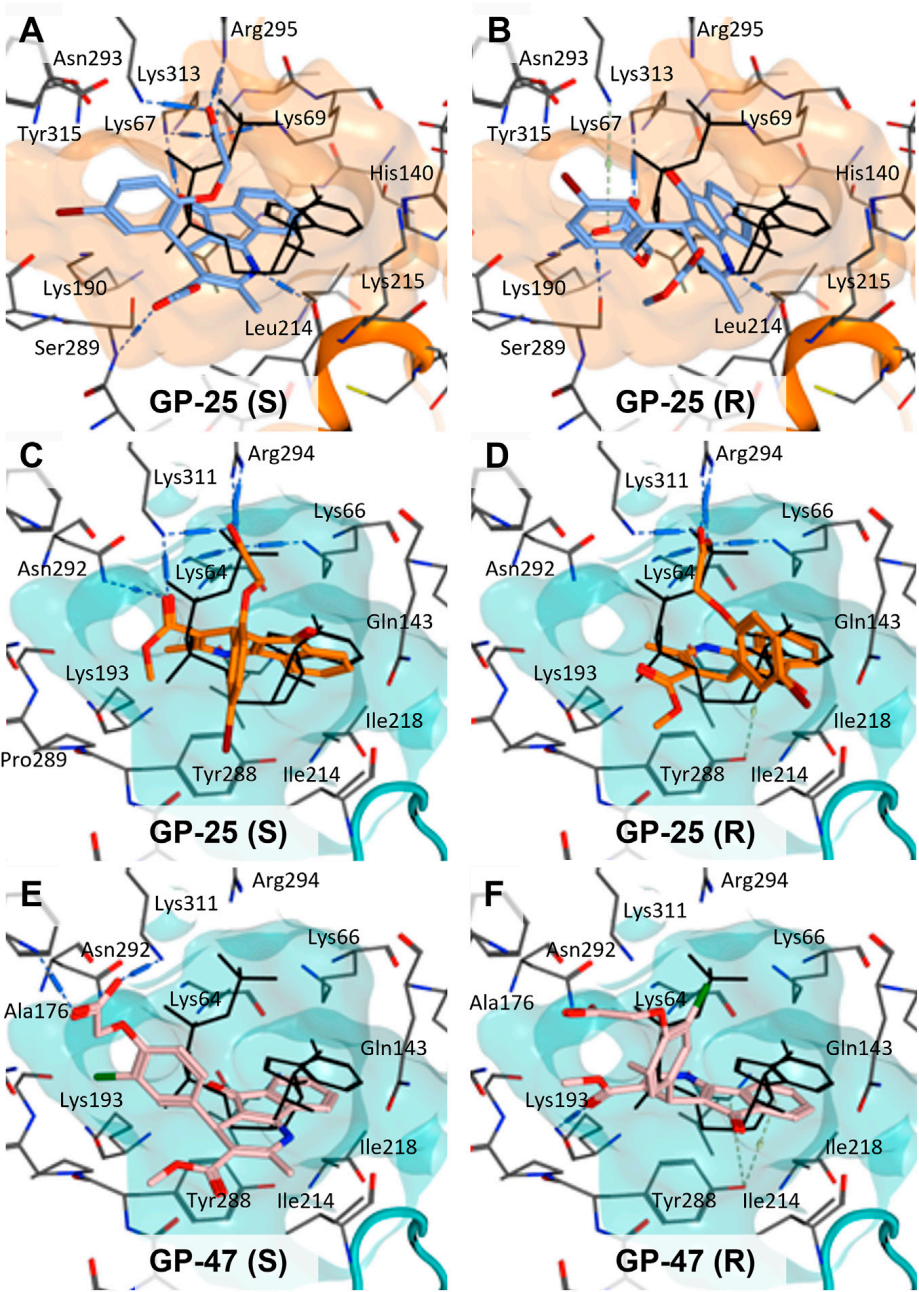
Docking of GP-25 and GP-47 into the orthosteric pockets of molecular models of human P2X4 and rat P2X7. **(A,B)** Docking of GP-25 (lilac) into human P2X4 (wheat). **(C,D)** Docking of GP-25 (orange) into human P2X7 (pale blue). **(E,F)** Docking of GP-47 (pale pink) into human P2X7 (pale blue). **(A,C,E)** represents the S-enantiomer and **(B,D,F)** represents the R-enantiomer. The ATP pose derived from the original crystal or cryoEM structure is shown in black lines for reference. Interactions are shown as dashed lines.

To investigate potential binding modes of GP-25 to P2X4 and P2X7, docking of the ligands was performed in the orthosteric pockets of our human P2X4 model (GP-25 was re-docked), and of a model of human P2X7 built from the cryoEM structure of rat P2X7 bound to ATP (PDB ID: 6U9W; McCarthy et al., 2019). Both GP-25 and GP-47 contain a chiral center and were purchased as mixture of enantiomers (no info on % composition provided by the supplier). Our docking investigated the binding mode of both enantiomers (S and R, Figure 5) in the orthosteric pocket using Glide Extra Precision (XP).

The carboxyl groups of the S-enantiomer of GP-25 form extensive interactions with many of the polar residues in a similar fashion to that of the gamma phosphate of ATP (Lys-67, Lys-69, Arg-295 and Lys-313 (human P2X4 numbering); upper part of Figure 5A). However, docking the R-enantiomer of GP-25 showed a flipped pose with the carboxyl moiety pointing toward the bottom of the pocket (lower part of Figure 5B), lacking the interactions with residues that coordinate the gamma phosphate in ATP. Conversely, for human P2X7, we were able to obtain (for both GP-25 enantiomers), poses where the carboxylic group made strong multiple interactions with polar residues known to interact with ATP in the crystal structure, specifically coordinating the gamma phosphate (Figure 5C, D), including Lys-311, Lys-64, Lys-66 and Arg-294 (human P2X7 numbering). Additionally, the hetero-tricyclic scaffold of GP-25 tightly occupies the cavity where the adenine ring of ATP is found in the cryoEM structure, unlike in human P2X4 (compare Figure 5C, D with Figure 5A, B). Docking of GP-47 into the orthosteric pocket of human P2X7 (Figure 5E, F) gave rise to poses where some interactions between the carboxylic acid moieties and key positively charged amino-acids (e.g. Lys-311 in the S-enantiomer; Figure 5E) were present, and the adenine pocket at least partially occupied by the tricyclic ring. The combination of lack of key interactions with conserved phosphate-coordinating amino-acids and lack of occupation of the adenine pocket for one of the two enantiomers may explain why GP-25 displays little antagonist activity at human P2X4 compared to human P2X7.

## 4 Discussion

In this work we have discovered a novel orthosteric P2X7 receptor antagonist using structure-based virtual screening, and screened analogues to analyze the molecular determinants of subtype-specific potency. Using a molecular model of human P2X4 based upon the ATP-bound *Danio rerio* crystal structure, we validated 3 separate docking algorithms by comparing the poses of docked ATP with the pose analogous to the crystal structure. We then performed a virtual screen, selecting the top 17 best-scoring compounds (according to a combination of the three algorithms) for functional assay. While none of the compounds in our initial screen displayed antagonist activity at human P2X4, several compounds were active at human P2X7, including GP-25, and we were able to confirm its antagonist activity in two separate assays (ATP-induced dye uptake and patch-clamp electrophysiology). Schild analysis suggested an orthosteric mode of action, with the caveat that the maximum response was reduced at the highest antagonist concentration tested. Screening a series of commercially available GP-25 analogues enabled the identification of several additional antagonists, and the development of a structure-activity relationship. Finally, docking of the two enantiomers of GP-25 and its close analogue GP-47 into models of human P2X4 and P2X7 enabled us to determine why GP-25 lacks activity at human P2X4, and why GP-25 and its analogues are more active at human P2X7 than rat P2X7.

The inter-subunit ATP-binding site of P2X receptors is highly conserved, and the overall shape and size of the ATP binding pocket is very similar between receptor subtypes (Kawate, 2017; Pasqualetto et al., 2018). Indeed, the binding pose of ATP in the pocket is strikingly similar in *Danio rerio* P2X4, human P2X3, gulf coast tick P2X and rat P2X7, adopting a U-shaped conformation where conserved lysine and arginine residues coordinate the phosphates. The presence of the gamma phosphate is crucial for agonist activity, as well as the conformation of the ribose moiety and the localization of the adenine moiety into a relatively hydrophobic pocket (Dal Ben et al., 2019; Gasparri et al., 2019; Grimes et al., 2020). We chose a model of human P2X4 based on the ATP-bound structure of *Danio rerio* P2X4 for virtual screening and validated our docking algorithms using ATP (in a model where the bound ATP was removed prior to docking). Overall, our algorithms replicated the pose of bound ATP quite well, giving us confidence that our virtual screening would yield molecules capable of docking to the orthosteric pocket.

Structure-based virtual screening has been used previously to discover novel P2X7 antagonists, using both the orthosteric (Caseley et al., 2016) and allosteric (Zhao et al., 2021) pocket. In both cases molecular models of human P2X7 based on homologous P2X receptor crystal structures were used and hit compounds with micromolar potency were obtained. This previous success with P2X7 models indicates that our strategy is valid, and a similar approach to ours has recently been carried out on a molecular model of human P2X4 (Beswick et al., 2019), discovering hit compounds, but with very low (high micromolar) potency. There has been a lower success with a similar approach for human P2X4 and the discovery of antagonists using the hP2X4 orthosteric pocket in virtual screenings has been more challenging. Indeed, in our study, multiple compounds initially selected as candidates from a P2X4 virtual screening displayed no activity at human P2X4, but instead displayed activity at human P2X7, including GP-25, which to our knowledge represents a novel P2X7 antagonist with no activity at human P2X4 receptors. A GP-25 analogue ((4-bromo-2-[3-(ethoxycarbonyl)-2-methyl-5-oxo-4,5-dihydro-1H-indeno[1,2 -b]pyridin-4-yl]phenoxy) acetic acid; Chembridge ID 6422575) has previously been shown to bind to the tyrosine kinase Syk with a Kd of 6.2 µM, and inhibition of antibody binding of 81% in an antibody displacement assay, but its IC_50_ for inhibiting mast cell degranulation (a key functional consequence of Syk activation) was ≥20 μM, suggesting a lack of biological effect, and no further studies were undertaken with this molecule (Villoutreix et al., 2011).

The fact that our virtual screen was performed on P2X4, yet we discovered compounds with activity at P2X7, seems surprising, and may appear to cast doubt on the value or validity of the virtual screening process. However, we suggest that the process was successful in part because of the high degree of similarity between the orthosteric pockets of P2X4 and P2X7, and in part because of the favorable binding of both enantiomers of GP-25 to the orthosteric pocket of P2X7 (see below). Screening GP-25 analogues, we found three (GP-47, GP-50, and GP-66) with similar activity to GP-25 at human P2X7 (but displaying no activity at the rat isoform), and confirmed a lack of cytotoxicity in both GP-25 and GP-47. Future studies could focus on chemical modification to these compounds to improve their potency, and experiments with other P2X7 subtypes to confirm selectivity.

The characterization of GP-25 included Schild analysis, which suggested a competitive (orthosteric) mode of action. However, the maximum concentration of GP25 that we could apply was 100 µM (due to solubility issues), and at this concentration we did observe a small reduction in maximum response. It is difficult to address this directly by making mutations in the orthosteric pocket, as these impair P2X receptor function (Chataigneau et al., 2013). Binding to the allosteric pocket could be confirmed or refuted in future experiments by assessing the ability of GP25 to antagonise receptors with mutations in the allosteric pocket, as demonstrated for the P2X7 antagonist AZ10606120 (Allsopp et al., 2017). This would not, however, rule out the possibility of binding at a distinct allosteric site. In future, direct structural study may be the only way to confirm the orthosteric binding of GP-25.

Given that GP-25 was initially selected in a screen against human P2X4, we considered potential reasons for its lack of activity at human P2X4 compared to human P2X7. GP-25 and its analogues have two possible enantiomers (R- and S-) due to the presence of a chiral center (Figure 4C, marked with a blue star) linking the heterotricyclic ring to the phenyl moiety, and we used molecular docking of both enantiomers of GP-25 into human P2X4 and P2X7, and both enantiomers of its analogue GP-47 into human P2X7, to try to address this. We found that, in human P2X4, only the S-enantiomer of GP-25 was able to make extensive interactions between its carboxylic acid moieties and the amino-acids known to coordinate the gamma phosphate of ATP, whereas in human P2X7, both enantiomers were capable of this. Furthermore, in human P2X4, the S-enantiomer of GP-25 made an additional stabilizing H-bond interaction with the backbone of Ser-289 and Leu-214 (the reason why it was chosen after visual inspection to be included in the initial screening). In the dock of the R-enantiomer of GP-25 into human P2X4, the carboxylic acid moieties were flipped towards the “bottom” of the orthosteric pocket, and while multiple interactions were observed, (a π-cation interaction with the phenyl moiety and Lys-313, and multiple H-bond interactions with Leu-214, Ser-289, Lys-67), the network of interactions was less complex than that of the S-enantiomer, and did not include Lys-69 or Arg-295. The lack of ability of the R-enantiomer of GP-25 to make interactions with the amino-acids that coordinate the gamma phosphate may explain the lack of activity at human P2X4. It is important to state that we do not know the proportions of R-and S-enantiomers present in the supplied compound; if it were a 50:50 mixture, according to our hypothesis above, we might have expected to observe some inhibition of P2X4 when 10 µM compound was applied, or inhibition at higher concentrations (e.g., 30–100 µM, which we did not test). However, if the R-enantiomer predominates, we would not necessarily expect to observe inhibition even at high concentrations. A possible reason why docking of the R-enantiomer of GP-25 resulted in a flipped conformation in P2X4 but not P2X7 is the reduced and more enclosed pocket in P2X4 compared to P2X7, due to the presence of a 4-amino-acid longer loop lining the side of the pocket (Pasqualetto et al., 2018).

In our docking of the S-enantiomer of GP-25 into human P2X7, the methoxycarbonyl moiety made further stabilizing interactions with Asn-292 and Lys-311. The necessity for there to be a non-planar dihedral angle between the ester and the Asn-292 and Lys-64 (to ensure optimal geometry for hydrogen bond formation) may at least partially explain that replacement of the freely rotating bond of the ester with a constrained cyclized carbonyl (e.g., in compound 18, 25, 26, 27, 28 or 30) always resulted in loss of activity. The hydroxyl group of Tyr-288 in human P2X7 also appears to be important for stabilizing the heterotricyclic ring of either GP-25 or GP-47. One of the very few differences between human P2X7 and rat P2X7 is the presence of a phenylalanine at position 288 (Phe-288), and weaker additional stabilization of GP-25 and its analogues in the orthosteric pocket may explain the moderate difference in potency of GP-25 at human over rat P2X7 and the loss of potency of GP-47 in rat P2X7.

In summary, we have discovered a novel P2X7 antagonist with an IC_50_ value of 8.7 μM at human P2X7, which displays no activity at human P2X4. Pharmacological and molecular docking analysis suggests that it may bind in the orthosteric pocket of the receptor, and that interactions between carboxylic acid moieties and positively charged amino-acids in the orthosteric pocket are potentially important for its activity and selectivity.

## Data Availability

The raw data supporting the conclusion of this article will be made available by the authors, without undue reservation.
