# Supplementary material for: Identification of a novel P2X7 antagonist using structure-based virtual screening
Source: Front Pharmacol. 2023 Jan 12;13:1094607. doi: 10.3389/fphar.2022.1094607 (PMC9877316; doi:10.3389/fphar.2022.1094607)
Supplement: Supplementary file 1 [file DataSheet1.docx]

Supplementary Material

#
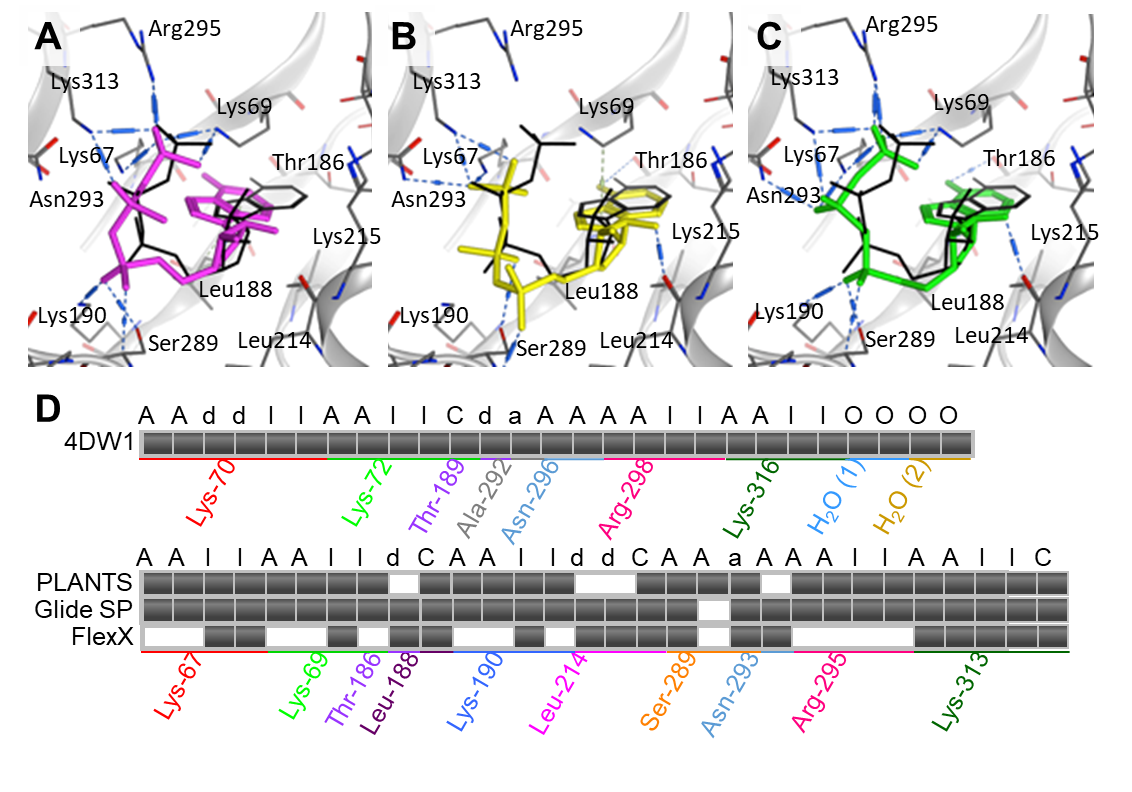
Supplementary Figure

**Supplementary Figure 1. Dock of ATP into the molecular model of human P2X4.** PLANTS (A, magenta), FlexX (B, yellow) and Glide SP (C, green) docking algorithms were validated by docking ATP in the human P2X4 model generated from 4DW1 (Hattori and Gouaux, 2012). H-bond interactions are shown with blue dotted lines and the conformation of ATP in the crystal structure (black) has been superposed for comparison. (D) Barcode plot representing the protein-ligand interaction fingerprint (PLIF) of the interactions formed by each docked pose (PLANTS, Glide SP, FlexX) in the human model compared to the ones detected in the crystal structure (4DW1). Grey-filled boxes indicate presence of the interaction type specified by the single letter (at the top) for the indicated residue (at the bottom). Same colour were used to label residues of the human model corresponding to the zebrafish orthologous. White boxes indicate no interactions. ‘A’, sidechain hydrogen bond acceptor; ‘a’, backbone hydrogen bond acceptor, ‘C’, surface contact, ‘d’, sidechain hydrogen bond donor; ‘I’, ionic attraction; ‘O’, solvent hydrogen bond.
